# Elevated blood levels of Dickkopf‐1 are associated with acute infections

**DOI:** 10.1002/iid3.232

**Published:** 2018-07-20

**Authors:** Melody Mazon, Valérie Larouche, Maryse St‐Louis, Detlev Schindler, Madeleine Carreau

**Affiliations:** ^1^ Centre Hospitalier de Québec‐Université Laval Research Center Québec G1V 4G2 Canada; ^2^ Department of Pediatrics Université Laval Québec G1V 0A6 Canada; ^3^ Research and Development Héma‐Québec Québec Canada; ^4^ Department of Human Genetics University of Wurzburg Wurzburg 97070 Germany

**Keywords:** Blood plasma, Dickkopf‐1, ELISA, Fanconi anemia, infections

## Abstract

**Introduction:**

Dickkopf‐1 (DKK1) is a soluble protein and antagonist of the Wnt/β‐catenin signaling pathway. DKK1 is found elevated in serum from patients affected with various types of cancers and in some instances, it is considered a diagnostic and prognostic biomarker. Elevated serum levels of DKK1 have also been detected in animal models of chronic inflammatory diseases. Previous work from our laboratory has demonstrated upregulation of DKK1 in cells and mouse models of the bone marrow failure (BMF) and cancer‐prone disease Fanconi anemia (FA). The present study aimed to investigate whether DKK1 blood levels in patients are associated with FA or inflammatory responses to acute infections.

**Methods:**

Plasma samples were collected from 58 children admitted to the Centre Mère‐Enfant Soleil du Centre Hospitalier de Québec‐Université Laval with signs of acute infections. Blood plasma specimens were also collected from healthy blood donors at the Héma‐Québec blood donor clinic. Plasmas from patients diagnosed with FA were also included in the study. DKK1 levels in blood plasmas were assessed by standard ELISA.

**Results:**

Patients with acute infections showed dramatically high levels of DKK1 (6072 ± 518 pg/ml) in their blood compared to healthy blood donors (1726 ± 95 pg/ml). No correlations were found between DKK1 levels and C reactive protein (CRP) concentration, platelet numbers, or white blood cell counts. Patients with FA showed higher DKK1 plasma levels (3419 ± 147.5 pg/ml) than healthy blood donors (1726 ± 95 pg/ml) but significantly lower than patients with acute infections.

**Conclusion:**

These findings suggest that blood DKK1 is elevated in response to infections and perhaps to inflammatory responses.

## Introduction

Dickkopf‐1 (DKK1) is a secretory protein and antagonist of the Wnt/β‐catenin signal pathway 1, 2. Activation of the Wnt/β‐catenin pathway induces expression of the *DKK1* gene. Production of DKK1 acts as a feedback mechanism to limit the Wnt/β‐catenin pathway activation. The ability of DKK1 to block Wnt/β‐catenin activity comes from its capability to interact directly with the Wnt co‐receptor LRP5/6 (low density lipoprotein receptor‐related protein 5 or 6) or indirectly by binding with its receptor Kremen‐1/2 and forming a ternary complex with LRP5/6 2, 3, 4, 5, 6, 7, 8. These interactions prevent the formation of an active Wnt‐Frizzled‐LRP5/6 complex. DKK1 plays fundamental roles in embryogenesis and is required for head induction, eye and limb formation, vertebral and bone development 2, 9, 10, 11, 12, 13. *DKK1* expression is high during development but is relatively low in most adult tissues. However, overexpression of *DKK1* is associated with several diseases that include various types of cancers 2, 14. Increased expression of *DKK1* is found in cancer cells, cancer surrounding tissues and elevated levels of DKK1 in peripheral blood are detectable in patients with cancers 15, 16. In fact, blood levels of DKK1 correlate in some cancers with prognosis 16, 17, 18, 19. Consequently, measurement of DKK1 in plasma or serum is viewed as a diagnostic and prognostic biomarker 20. Moreover, elevated levels of DKK1 in peripheral blood are associated with chronic inflammatory diseases 21.

Interestingly, we previously reported overexpression of *DKK1* in cells derived from Fanconi anemia (FA) patients and elevated levels of Dkk1 in blood of FA mutant mice 22. FA is a BMF syndrome associated with congenital malformations and cancer predisposition 23, 24. FA is associated with 22 subtypes (FANC‐A to W) and characterization of the related FA genes has led to the identification of a molecular pathway known as the FA pathway 25, 26. This pathway is a guardian of genome integrity during cellular division 26. In addition, several FA proteins act in other cellular functions including regulation of transcription, response to viral infections and oxidative stress 23. Physiological stresses such as infection‐associated inflammation in FA mutant mice lead to BMF and in part recapitulate the human disease FA 27, 28.

Given that DKK1 is dysregulated in cells and mouse models of FA, that inflammation in FA leads to BMF and that DKK1 is activated in response to inflammation, we hypothesized that DKK1 levels increase in response to infections with or without accompanying inflammation. We thus evaluated DKK1 levels in peripheral blood from children affected by acute infections in comparison to patients with BMF including FA.

## Methods

### Study design and patients

Children admitted to the Centre Mère‐Enfant Soleil du Centre Hospitalier de Québec‐Université Laval (CHU) with signs of acute infections were recruited and included in the study. Inclusion criteria consisted of patients aged 1 month to 17 years showing signs of infections. Exclusion criteria comprised patients suffering from cancer, anemia, or any other hematological abnormalities. Complete blood counts and CRP levels were analyzed as part of the clinical evaluation. Informed consent was obtained from each patient or parent. The study protocol was approved by the CHU Ethical review board. Blood plasma from healthy donors (Controls) were obtained from the Héma‐Québec blood donor clinics after informed consent according to Héma‐Québec guidelines. Plasma samples previously obtained from patients with BMF that were subsequently diagnosed with FA or excluded from FA (BMF) were collected over several years from Germany patients within the framework of FA diagnostics following informed consent and approval by the Institutional Ethical review boards.

## ELISA

Plasma from patients and donors was subjected to an enzyme‐linked immunosorbent assay (Human DKK‐1 Quantikine ELISA kit, DKK100; R&D Systems, Minneapolis, MN) to evaluate DKK1 concentration according to the manufacturer's instructions. Each sample was analyzed in duplicate.

### Statistical analyses

Sample size calculation was performed to obtain significant differences in DKK1 levels between populations with a power of >0.8 and a *p* = 0.05 with a minimum of 50 subjects per group. DKK1 levels were expressed as the means ± standard errors of the mean (SEM). Statistical analyses were performed with GraphPad Prism software (La Jolla, CA, USA) (version 5.0b), and the tests used included linear regression, Pearson's correlation, and two‐tailed Student's *t*‐tests. Results with a *p* values less than 0.05 were considered significant.

## Results

### DKK1 overproduction in children with infectious diseases

**Figure 1:**
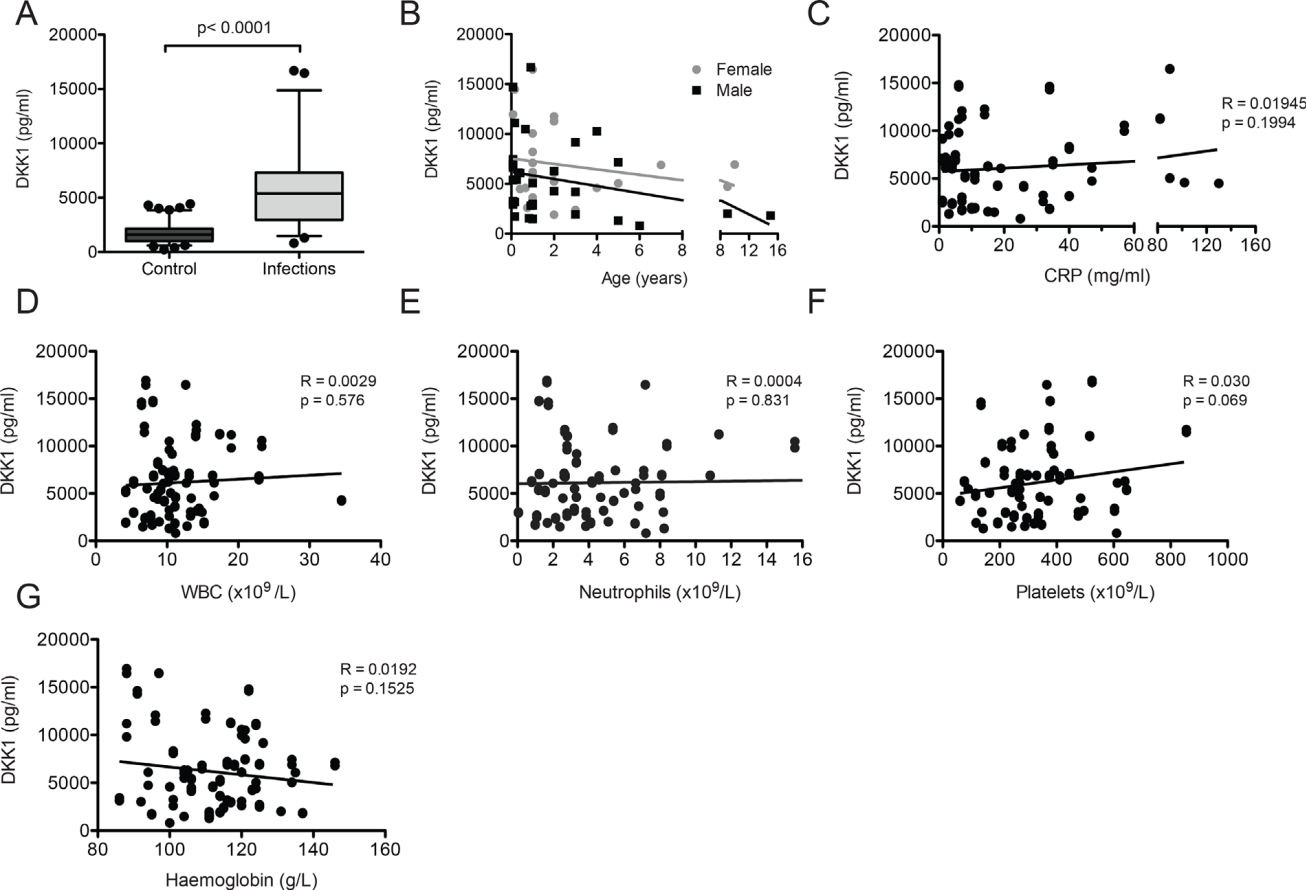
DKK1 levels in blood from children with acute infections. (A) DKK1 plasma levels from children suffering from various infections (*n* = 57) and from healthy blood donors (Control, *n* = 107). Graphs represents the average of two separate determinations for each patient's sample. Statistical significance was determined by unpaired Student *t*‐test. (B‐G) DKK1 levels from patients with infections according to (B) age and gender, (C) levels of the C‐reactive protein (CRP), (D) white blood cell counts (WBC), (E) neutrophils, (F) platelets, and (G) hemoglobin. Statistical significance was determined by linear regression (*R* ^2^) and Pearson's correlation.

Blood plasma was collected from a total of 57 children, 33 males, and 24 females, aged 1 month to 15 years (Table 1). These patients suffered from the different infections listed in Table 2. Plasma DKK1 levels were analyzed from blood samples obtained at the time of admission as part of the clinical evaluation. DKK1 levels in patients with acute infections were found dramatically elevated (mean of 6072 ± 518 pg/ml) compared with 107 healthy blood donors (1771 ± 95 pg/ml; Fig. 1A). No significant correlations were observed between levels of DKK1 and age, gender, levels of CRP, white blood cell counts, neutrophils, platelets, or haemoglobin (Fig. 1B–G). Also, the type of infection did not seem to influence DKK1 production, suggesting that no specific pattern‐recognition receptors critical for the host defence system are involved.

**Table 1:** DKK1 levels in blood of patients with infections or hematological disorders

Variables	Control	Infections	FA	BMF
Number of patients	107	57	98	58
Males	54	33	56	26
Females	53	24	42	32
Age at draw (range in years)	18–45	0.08–15	0.08–37^a^	0.08–64
DKK1 values (pg/ml)				
Mean	1771	6072	3465	4575
Median	1595	5391	3212	4134
SD	979	3912	1888	2755

SD, standard deviation.

^a^ Seven patients with missing age at draw.

**Table 2:** DKK1 levels in children with infectious diseases

	DKK1 levels^a^	
Variables	High (>5391 pg/ml)	Low (<5391 pg/ml)
No Patients	29	28
Male/female	16/13	17/11
Age groups		
>1 yr	14	9
≤1 yr	15	19
Types of infections		
Bronchiolitis	8	3
Hyperthermia/fever	2	6
Gastroenteritis	2	–
Pneumonia	8	7
Viral infection^b^	4	3
Cellulitis	1	2
Pyelonephritis	–	3
Sepsis	1	1
Adenitis	1	2
Skin abscess	1	–
Mononucleosis	1	
Otitis	–	1

^a^ High and low DKK1 levels based on one SD.

^b^ Viral infections: undefined viral infections, parotitis, upper respiratory tract infections of unknown origin.

### Patients with Fanconi anemia show elevated levels of DKK1

**Figure 2:**
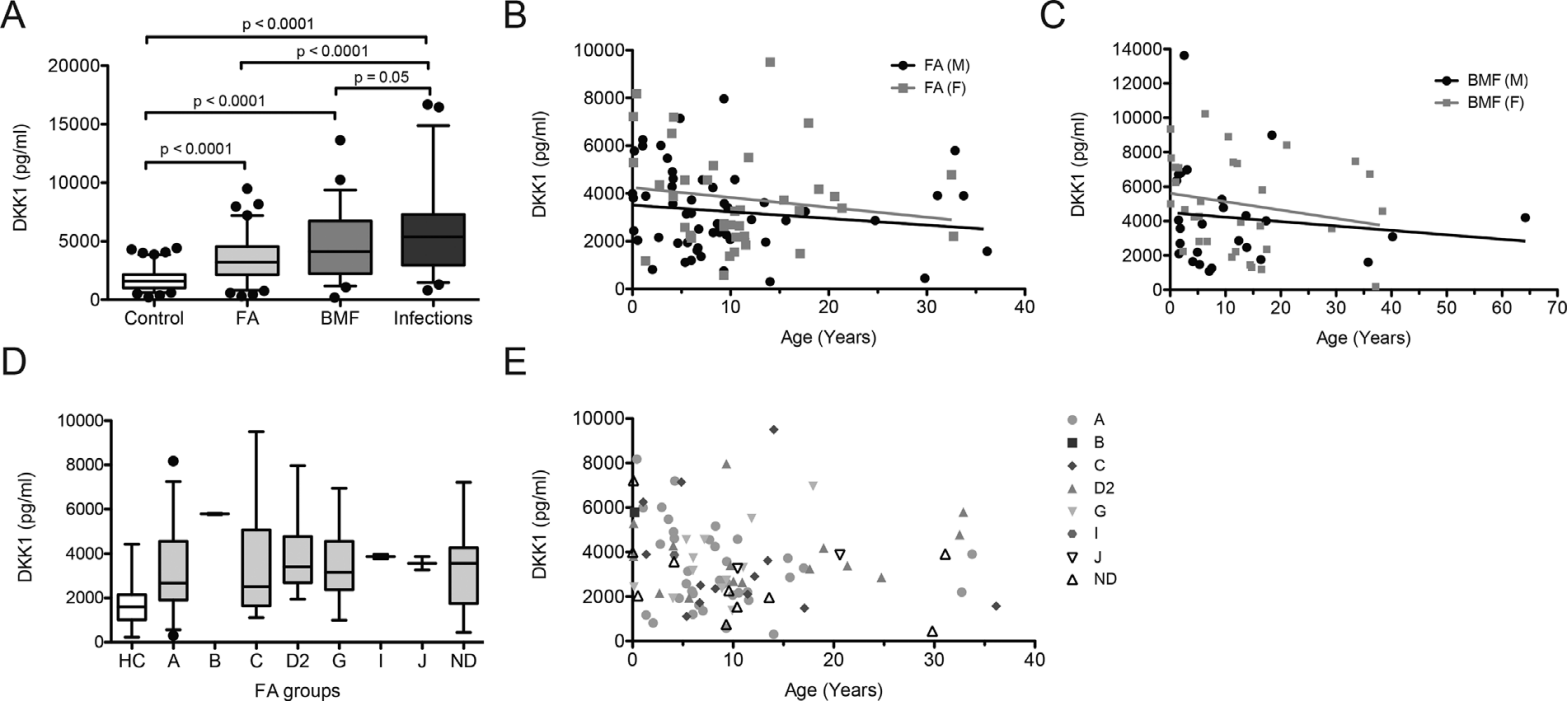
DKK1 levels in blood from patients with hematological disorders. (A) DKK1 plasma levels from patients diagnosed with FA (*n* = 98) or excluded from FA (BMF, *n* = 58) and as in Fig. 1 and from children suffering from various infections (*n* = 57) and from healthy blood donors (Control, *n* = 107). BMF represent patients with bone marrow failure presented as severe aplastic anemia or myelodysplasia that were excluded from FA at the time of diagnosis. Graphs represents the average of two separate determinations for each patient's sample. (B and C) DKK1 levels from FA (B) and BMF (C) patients according to age and gender. (D and E) DKK1 levels according to the FA gene mutated (in D) and age (in E). HC, healthy controls; ND, not determined. Statistical significance was determined by unpaired Student *t*‐test, linear regression (*R* ^2^) and Pearson's correlation.

Peripheral blood plasmas were obtained from a total of 98 patients with FA, both males and females, aged 1 month to 37 years (56 males, 42 females), and 58 patients with BMF (26 males, 32 females; aged 1 month to 64 years) but excluded from FA (Table 1). Patients found positive for FA were assigned to complementation groups A, B, C, D2, G, I, or J. Fifteen patients diagnosed with FA but with undetermined mutations at the time of diagnosis and seven patients with FA with missing age at draw were also included in the study. DKK1 levels found in patients with FA were compared to those found in patients with acute infections and healthy donors. Results show that patients with FA presented with elevated DKK1 levels in their blood (mean value of 3465 ± 190 pg/ml) compared to healthy blood donors (1771 ± 95 pg/ml) but significantly less than patients with acute infections (mean value of 6072 ± 518 pg/ml; Table 1 and Fig. 2). DKK1 levels were similar whether blood was collected onto heparin, EDTA, or sodium citrate (data not shown) as previously reported 29. We also evaluated DKK1 levels in plasma samples obtained from 58 patients admitted on the basis of BMF. Those patients were subsequently excluded from the diagnosis of FA and included 26 males and 32 females aged 1 month to 64 years (Table 1). BMF patients presented with a significant increase in DKK1 protein levels in their blood (4575 ± 362 pg/ml) compared with healthy blood donors. Surprisingly, BMF patients presented significantly more elevated levels of DKK1 than patients with FA but lower than children with acute infections (Table 1 and Fig. 2A). Statistical analysis showed no correlation between DKK1 levels and patient's age or gender in FA and BMF populations (Fig. 2B and C). In addition, no correlations were found between DKK1 levels and the FA gene mutated (Fig. 2D and E). These results suggest that patients with BMF or FA present elevated levels of DKK1 in their blood.

Together, our results suggest that the presence of elevated DKK1 levels in peripheral blood is indicative of inflammatory or stress signals such as marrow failure.

## Discussion

Identification of disease biomarkers is of importance for early interventions, to monitor disease progression or to evaluate treatment responses. DKK1 has been proposed as a potential biomarker for cancer progression and prognosis. Elevated blood levels of DKK1 have been associated with multiple myeloma and various types of cancers including head and neck, lung, breast, liver, and bone cancers 20. Given that elevated levels of DKK1 were found in blood of FA‐deficient mice and that FA is a cancer prone disease, DKK1 might be of interest for FA. In fact, studies with similar methods of detection show comparable levels of DKK1 between plasma from patients with hepatocellular carcinoma (mean of 3400 pg/ml) 18 to those from patients with FA (mean of 3465 pg/ml; our study) suggesting that increased DKK1 in patients with FA might reflect a propensity for cancer. However, based on the present study showing that acute infections trigger overproduction of DKK1, elevated levels of DKK1 in the blood of patients with FA may reflect the presence of an inflammatory or stress response rather than cancer. This may also be true for patients diagnosed with cancers. Actually, the *DKK1* gene was shown to be activated in response to inflammatory and stress signals and the Dkk1 protein was found elevated in blood of animal models of inflammation and radiation‐induced stress 20, 30, 31, 32, 33, 34, 35. These findings support our data and suggest that DKK1 activation and overproduction might be indicative of inflammatory responses in patients rather than malignancies per se.

Surprisingly, but consistent with previous reports, the levels of DKK1 did not correlate with levels of the CRP, which is an acute‐phase marker of inflammation 36, 37. While CRP is produced by hepatocytes in response to cytokines produced during an acute‐phase event 38, the site of DKK1 production remains to be identified. Previous reports have suggested that even though DKK1 is not produced by platelets, it may be stored in platelets and released upon activation 29, 31. In our study, we did not observe any correlation between the number of platelets and DKK1 levels in blood from children with infectious diseases. Unfortunately, we do not have platelet counts from the FA and BMF populations included in this study. Because thrombocytopenia is a feature of FA, we could argue against a role of platelets in DKK1 overproduction at least in these patients.

The strengths of our study reside in the number of samples obtained and the wide range in age at diagnosis for patients with FA or excluded from FA and patients with acute infections. The limitations of our study include differences in age distribution between healthy donors and patients. However, DKK1 levels were not influenced by age nor gender in the different populations. Another limitation is the lack of clinical data in the FA and BMF cohorts and follow‐up of patients with infections. Although the heterogeneity of infections may be interpreted as a limitation of our study, the fact that both high (over one SD) and low (below one SD) DKK1 levels were found within each type of infections indicate that inflammatory responses induce DKK1 overexpression regardless of the type of pathogen.

In summary, the association between DKK1 and acute infections in our study is a novel observation. Based on our data, we advise caution for the use of DKK1 blood levels as an indicator of the course or prognosis of cancer or chronic diseases in patients. However, we propose that DKK1 may serve as an indicator of inflammatory responses that could complement other biomarkers of disease progression. Further testing will be important to determine the actual mechanism leading to increased DKK1 production during infections and whether DKK1 is a marker of chronic or undetected infections secondary to other diseases such as FA.

## Conflict of Interest

The authors declare having no conflict of interest.
